# Primary human nasal epithelial cell response to titanium surface with a nanonetwork structure in nasal implant applications

**DOI:** 10.1186/s11671-015-0849-8

**Published:** 2015-04-08

**Authors:** Wei-En Yang, Ming-Ying Lan, Sheng-Wei Lee, Jeng-Kuei Chang, Her-Hsiung Huang

**Affiliations:** Institute of Oral Biology, National Yang-Ming University, No.155, Sec.2, Linong Street, Taipei, 112 Taiwan; Department of Otolaryngology, Taipei Veterans General Hospital, No.201, Sec.2, Shipai Road, Taipei, 112 Taiwan; School of Medicine, National Yang-Ming University, No.155, Sec.2, Linong Street, Taipei, 112 Taiwan; Institute of Materials Science and Engineering, National Central University, No. 300, Jhongda Road, Taoyuan, 320 Taiwan; Department of Dentistry, National Yang-Ming University, No.155, Sec.2, Linong Street, Taipei, 112 Taiwan; Graduate Institute of Basic Medical Science, China Medical University, No.91, Hsueh-Shih Road, Taichung, 404 Taiwan; Department of Medical Research, China Medical University Hospital, No.2, Yude Road, Taichung, 404 Taiwan; Department of Biomedical Informatics, Asia University, No.500, Lioufeng Road, Taichung, 413 Taiwan; Department of Stomatology, Taipei Veterans General Hospital, No.201, Sec.2, Shipai Road, Taipei, 112 Taiwan

**Keywords:** Human nasal epithelial cell, Titanium nasal implant, Electrochemical anodization, Nanonetwork, Cell response

## Abstract

In nasal reconstruction applications, the response of cells to titanium (Ti) implants is largely determined by the surface characteristics of the implant. This study investigated an electrochemical anodization surface treatment intended to improve the response of primary human nasal epithelial cells (HNEpC) to Ti surfaces in nasal implant applications. We used a simple and fast electrochemical anodization treatment, i.e., applying anodic current, to produce a titanium dioxide (TiO_2_) nanonetwork layer on the Ti surface with average lateral pore size below 100 nm, depending on the current applied. The TiO_2_ nanonetwork layer exhibited enhanced hydrophilicity and protein adsorption ability compared with untreated Ti surfaces. In addition, the spreading morphology, cytoskeletal arrangement, and proliferation of HNEpC on the nanonetwork layer indicated excellent cell response characteristics. This research advances our understanding regarding the means by which a TiO_2_ nanonetwork layer can improve the response of HNEpC to Ti surfaces in nasal implant applications.

## Background

Implants have a long history of being used in nasal surgery. In general, they are simply placed under the relatively thin skin or nasal mucosa as supportive materials to replace nasal bone or cartilage. Nasal implants may be used in various nasal surgeries, such as in the repair of nasal septal perforation [1-3], cerebrospinal fluid (CSF) rhinorrhea [4], and rhinoplasty [5].

Nasal septal perforation can vary in both size and location. The closure of nasal septal perforation is still a challenging problem for otolaryngologists. Small perforations do not require surgery, with the symptoms being alleviated using antibiotic ointment or a polymeric silicone button shaped to the individual perforation. For larger perforations, the use of an advanced nasal septum mucosal flap, inferior turbinate flap, or the selection of an ideal implant to repair the perforation may be considered.

Rhinoplasty, one of the most common plastic surgeries, requires implant usage in many situations, especially for nasal augmentation and reconstruction. The most common site requiring augmentation is the nasal dorsum, which may be congenitally low or malformed due to prior surgery or trauma. Various materials have been used in rhinoplastic surgery. The ideal implant materials should be readily available, inexpensive, inert, nontoxic, noncarcinogenic, sterilizable, easy to sculpt, easily camouflaged, and able to provide volume and mechanical support. Furthermore, the ideal implant should interact favorably with the surrounding tissues; maintain its form over time; resist trauma, infection, and extrusion; and remain easy to remove.

Titanium (Ti) metal has often been applied in the design of implants because of its favorable mechanical properties and biocompatibility. Using Ti nasal implants and the open rhinoplasty approach to repair large septal perforations has produced good results. Ti nasal implants can be used in both septal perforations and rhinoplasty. A previous study reported on the use of nasal implants as the nasal scaffolding in total nasal reconstructions in patients with nasal cavity cancer, which produced excellent cosmetic results [6]. Nasal implants have been reported to be capable of resisting contraction forces better than bone or cartilage, especially in patients who have undergone postoperative radiotherapy [6]. Moreover, a Ti nasal implant can be molded into a desired shape, acting as a thin but strong supporting bridge in rhinoplasty [5,7,8]. The implant can be placed into the nose through a tiny incision in the nostril [5,9]. However, as for all other types of implants, the possibility of implant extrusion still exists.

Ti implant has been used in nasal reconstruction applications. Literature on the clinical design and operation of Ti nasal implant has been reported [5,10]. It is well know that the biocompatibility of implant surface plays an important role in clinical success. However, the information on the biological response to Ti nasal implant surface is still very limited. The Ti metal surface spontaneously forms a protective TiO_2_ layer under atmospheric conditions. The surface characteristics of the TiO_2_ layer on the Ti surface determine the biocompatibility of the Ti implant. Surface topography is a key factor that regulates the responses of a cell to biomaterials, including cell adhesion, spreading, migration, proliferation, and differentiation [11-14]. Various surface modifications have been used in an effort to improve the interfacial properties between human cells and Ti-based implant surfaces [15-18]. Although Ti is extensively used in dental and bone implants, its application in nasal surgery is still rare.

In this study, a fast and simple electrochemical anodization treatment was used to produce a nanonetwork Ti-oxide layer on a Ti surface. Our hypothesis is that a nanoscale oxide layer on a Ti surface improves protein adsorption and human nasal epithelial cell responses. This nanonetwork structure on Ti surfaces may have potential in nasal implant applications.

## Methods

### Materials preparation

Commercial purity grade 2 Ti (diameter of 15 mm; thickness of 1 mm) specimens were used. The specimens were ground using SiC paper until #1200 and designated M. An electrochemical anodization treatment in 5 M NaOH solution (J.T. Baker, Avantor Performance Materials, Center Valley, USA) using two different anodic currents of E1 and E2 amperes (E1 < E2 < 0.5 A) was used to create the modified surface on the ground Ti surface. The corresponding specimens were designated E1 and E2. The total electrochemical anodization time was less than 1 h.

### Surface characterizations

Surface topography of the specimens was analyzed using field emission scanning electron microscope (FE-SEM). Surface roughness *R*_a_ of the specimens was analyzed using atomic force microscopy (AFM) with scanning area of 50 μm × 50 μm. Surface hydrophilicity, or wettability, was analyzed by measuring the contact angle of phosphate buffered saline (PBS; purchased from Sigma-Aldrich, St. Louis, USA) on the specimens using contact-angle goniometer. All measurements were performed in triplicate.

### Cytotoxicity assay

To assess the cytotoxicity of the test specimens in accordance with ISO 10993–5 standards [19], we obtained L929 mouse fibroblast cells from the Food Industry Research and Development Institute, Taiwan. The specimens were immersed in Eagle’s minimal essential medium (MEM; purchased from Sigma-Aldrich) at 37°C in a 5% CO_2_ incubator for 1 day to produce the extracts from the test specimens. The extract-containing media were then used to treat cell monolayers for 1 day, after which we investigated the cytotoxicity using a 3-(4,5-dimethylthiazol-2-yl)-2,5-diphenyl tetrazolium bromide (MTT; purchased from Sigma-Aldrich) assay and a microplate photometer (wavelength = 570 nm) to measure the optical density (OD). Higher OD values indicated greater cell viability. For this analysis, cells cultured in MEM without extract was used as blank group (blank); cells cultured in MEM with 10% dimethyl sulfoxide (Sigma-Aldrich) served as positive control (PC); and cells cultured in MEM with extract from zirconia served as negative control (NC).

### Protein adsorption

The specimens were immersed in a protein (bovine serum albumin (BSA) or fibronectin, purchased from Sigma-Aldrich) solution (5 mg/ml) at 37°C for 5 min. After the immersion process, the samples were washed with PBS and air dried. The adsorbed protein on the Ti surfaces was qualitatively analyzed using X-ray photoelectron spectroscopy (XPS) for depth profiling of N1s (at %) at 5, 15, and 30 s of sputter time.

### Cell response

The test specimens were sterilized using 15 W ultraviolet light with a distance of approximately 30 cm for 30 min before the following biological tests were conducted. The cell responses investigated in this study included cell adhesion and cell proliferation. Primary human nasal epithelial cells (HNEpC) were purchased from PromoCell (C-12620, Heidelberg, Germany) and cultured in an airway epithelial cell growth medium (C-21060, PromoCell) using a penicillin-streptomycin amphotericin B solution (Biological Industries, Kibbutz Beit-Haemek, Israel). The cells were maintained in a humidified incubator at 37°C with 5% CO_2_.

Crystal violet staining, FE-SEM micrograph, and immunofluorescent staining were used to study cell adhesion on the specimens. The HNEpC were cultured onto the specimens (1 × 10^4^ cells/cm^2^) in an incubator with 5% CO_2_ content at 37°C. The HNEpC were cultured on the specimens for 12 h. For the crystal violet staining, the cells were fixed using methanol (J.T. Baker), and 1% crystal violet (Showa, Tokyo, Japan) was used to stain the cells on the Ti specimens. An optical microscope was then used to observe the number of cells and cell spreading. For the FE-SEM observation, the cells were fixed using 4% paraformaldehyde (Sigma-Aldrich) and were dehydrated using a sequential series of ethanol (30% to 100%) (J.T. Baker). The cell adhesion morphology was analyzed using FE-SEM after coating the Ti specimens with a thin platinum film. For the immunofluorescent staining, after the cells were fixed with 4% paraformaldehyde (Sigma-Aldrich) and permeabilized with 0.3% Triton X-100 (J.T. Baker), diamidino-2-phenylindole (DAPI; Sigma-Aldrich) and rhodamine phalloidin (Biological Industries) were used to stain the nuclei (blue color) and actin filaments (red color), respectively. The images of the immunofluorescence-stained HNEpC were taken using a fluorescent microscope to analyze the cell adhesion morphology and cytoskeletal arrangement.

For the cell proliferation assay, a MTT (Sigma-Aldrich) assay was used to measure the cell viability at different cell incubation times. The HNEpC were seeded onto the Ti specimens (1 × 10^4^ cells/cm^2^) for 2, 4, and 6 days. After MTT treatment in a culture medium at 37°C for 4 h, the formazan was dissolved using isopropanol (Sigma-Aldrich) and measured spectrophotometrically at 570 nm. A minimum of three samples was used for each test group. Statistical analysis was used to evaluate the proliferation assay results. A significant difference from the respective controls was analyzed by Student’s *t* test for each paired experiment. A *p* value of <0.05 was regarded as a significant difference.

## Results and discussion

### Surface characterizations

**Figure 1:**
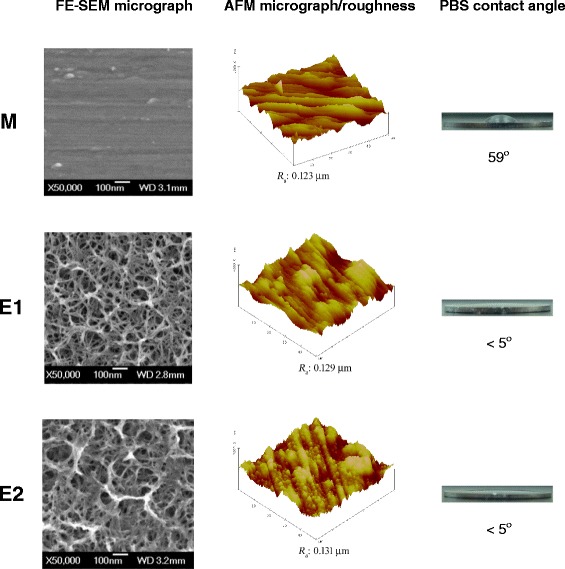
**FE-SEM micrographs of the non-anodized (M) and anodized (E1 and E2) Ti specimens.** FE-SEM surface micrographs, AFM 3D surface topography/roughness, and PBS contact angle of the Ti specimens with (E1 and E2) and without (M) electrochemical anodization.

The FE-SEM micrographs of the non-anodized (M) and anodized (E1 and E2) Ti specimens are presented in Figure 1. A unique nanonetwork structure with mixed pore sizes is observed on the anodized Ti specimen surfaces; this network structure is identified as anatase phase TiO_2_ [20]. The lateral pore size of the network on the E1 and E2 specimens varied from a few nanometers to the submicron range (approximately 10 to 150 nm). The average lateral pore size of the network was approximately 70 nm and 90 nm for the E1 and E2 specimens, respectively. According to our previous study, the cross-sectional thickness of the nanonetwork layer is approximately 200 nm for the E1 specimen and 300 nm for the E2 specimen [21]. The anodic current applied to the E2 specimen was higher than that applied to the E1 specimen during the anodization treatments, implying that the surface of the E2 specimen had a higher anodic dissolution rate than that of the E1 specimen. This explained why the E2 specimen presented a larger network pore size than the E1 specimens. Furthermore, it is crucial that nasal implants should provide enough mechanical stability despite their millimeter-scale thickness. The relatively thin nanonetwork layer (thickness 200 or 300 nm) on the anodized Ti specimen (thickness 1 mm) in this study is believed to have no negative effect on the mechanical properties of the Ti substrate in nasal implant applications. Thus, the primary effect of the surface nanonetwork structure is to enhance the biological response to the implant.

Figure 1 also shows the surface 3D topography/roughness and hydrophilicity of test specimens analyzed using AFM and contact-angle goniometer, respectively. The AFM analysis results revealed no notable differences in surface roughness *R*_a_ (0.123 to 0.131 μm) among the M, E1, and E2 specimens. The E1 and E2 specimens presented an *R*_a_ value only a few nm greater than that of the M specimen. The hydrophilicity analysis, which was obtained by measuring PBS contact angle, showed that the E1 and E2 specimens had a contact angle of <5°, whereas the M specimen presented a contact angle of approximately 59°. The electrochemical anodization process produced a surface nanotopography with super hydrophilicity but did not alter the surface roughness. These results suggested that the nanoscale proteins in the body environment are expected to easily penetrate the hydrophilic nanoporous structure on the E1 and E2 surface and assist the following cell response, as will discuss later.

Surface topography is known to influence cell responses [22]. Various cells are able to detect changes in surface topography, and much research has been aimed at the study of grooves of varying dimensions [23]. Surface roughness is a factor known to influence cell response. Extensive research on the production of materials with suitable roughened surfaces has therefore been conducted in an attempt to control cell responses to potential implant materials [24-30]. However, in this study, the manufacturing process of the anodized surfaces did not significantly affect the surface roughness but instead formed a very hydrophilic nanonetwork layer on the Ti surface. The surface chemical composition of the E1 and E2 specimens is primarily TiO_2_ [20], which is basically the same as the oxide that forms spontaneously on the M specimen in an air environment. This led us to the conclusion that the chemical composition on the surface did not have a significant effect on the hydrophilicity of the test specimens. The HNEpC response to the anodized Ti surfaces was mainly affected by the structure of the nanonetwork layer and its hydrophilicity. This unique super hydrophilic nanostructure was formed on the Ti surfaces in less than 1 h using a simple electrochemical anodization treatment. The time saving and low cost of this surface modification may be of interest in nasal implant applications.

### Cytotoxicity assay

**Figure 2:**
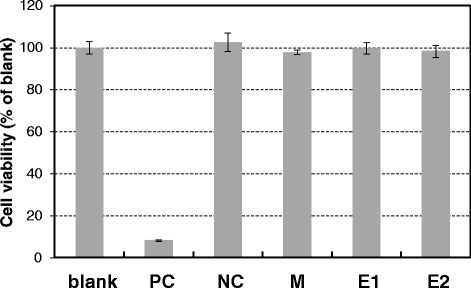
**Viability of L929 cells after being cultured in MEM with extracts from the test specimens.** ISO 10993–5 cytotoxicity assay results of the Ti specimens (M, E1, and E2), showing the viability of L929 cells after 1 day of incubation in extract-containing medium.

Figure 2 illustrates the viability of L929 cells after being cultured in MEM with extracts from the test specimens for a period of 1 day. As can be seen, the viability of cells cultured in extract-containing medium (M, E1, and E2) was on par with that of cells cultured in extract-free medium (blank). These results demonstrate the non-cytotoxicity of the M, E1, and E2 specimens.

### Protein adsorption

**Figure 3:**
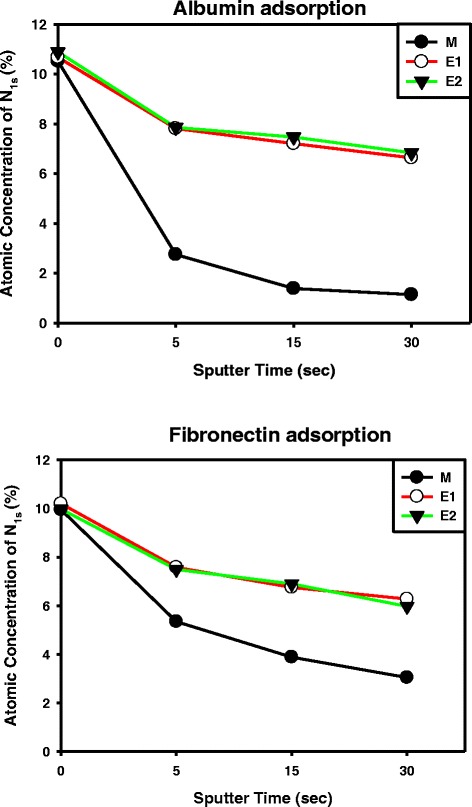
**XPS surface analysis results.** Depth profiling of N1s (atomic concentration), revealing the protein adsorption of the Ti specimens with (E1 and E2) and without (M) electrochemical anodization treatment.

XPS surface analysis was used to produce depth profiles of N1s (at %) using 5, 15, and 30 s of sputter time (Figure 3). As observed in Figure 3, albumin and fibronectin adsorbed onto the anodized E1 and E2 specimens. After 5 min of immersion in the albumin and fibronectin solution, the anodized E1 and E2 specimens exhibited higher N1s content (two- to fourfold) than the M specimen after 30 s of sputtering. The results revealed that the anodized E1 and E2 specimens were characterized by higher protein adsorption than the M specimen.

The serum protein covered the implant surface immediately when the biomaterial was brought into contact with blood. The adsorption of protein can provide adhesion sites for cell bonding to the surface, which can lead to additional cell behavior [31]. The adsorption of plasma proteins to the surface of a biomaterial is the first and most important event prior to the initiation of key cellular activities such as cell attachment, migration, differentiation, and proliferation.

Two type of proteins, serum albumin (non-cell adhesive) and plasma fibronectin (cell adhesive) [32], were used to investigate the protein adsorption ability of the Ti specimens. The first proteins to be adsorbed were the relatively abundant plasma proteins, such as albumin, fibrinogen, immunoglobulin G, and fibronectin. Fibronectin is a glycoprotein found in both the serum and extracellular matrix and is responsible for key functions including cell attachment and migration, as well as cell-cell and cell-substrate adhesion through integrin receptors [33]. In addition, fibronectin is a flexible protein that can adopt many conformations depending on the morphology of the surface, with its dimensions varying from an average length of 15 nm to 60 nm [34,35]. Albumin, a globular protein, has smaller dimensions (4 nm × 4 nm × 4 nm) [36] than fibronectin. The pore size range of the hydrophilic nanonetwork structure on the anodized Ti specimens covered the dimensional range of the albumin and fibronectin. This partially explained the fact that the anodized specimens adsorbed higher proteins than the non-anodized specimens. In addition, it is expected that the increase in surface area and the super hydrophilicity of the nanonetwork structure on the anodized specimens played a positive role in the promotion of protein adsorption. Proteins at the nanoscale level can sense the nanoscale topography, which induces subsequent cell responses, such as cell adhesion and cell spreading. In this study, a higher degree of protein adsorption on the anodized E1 and E2 specimens may further influence cell adhesion and cell proliferation.

### Responses of human nasal epithelial cells

**Figure 4:**
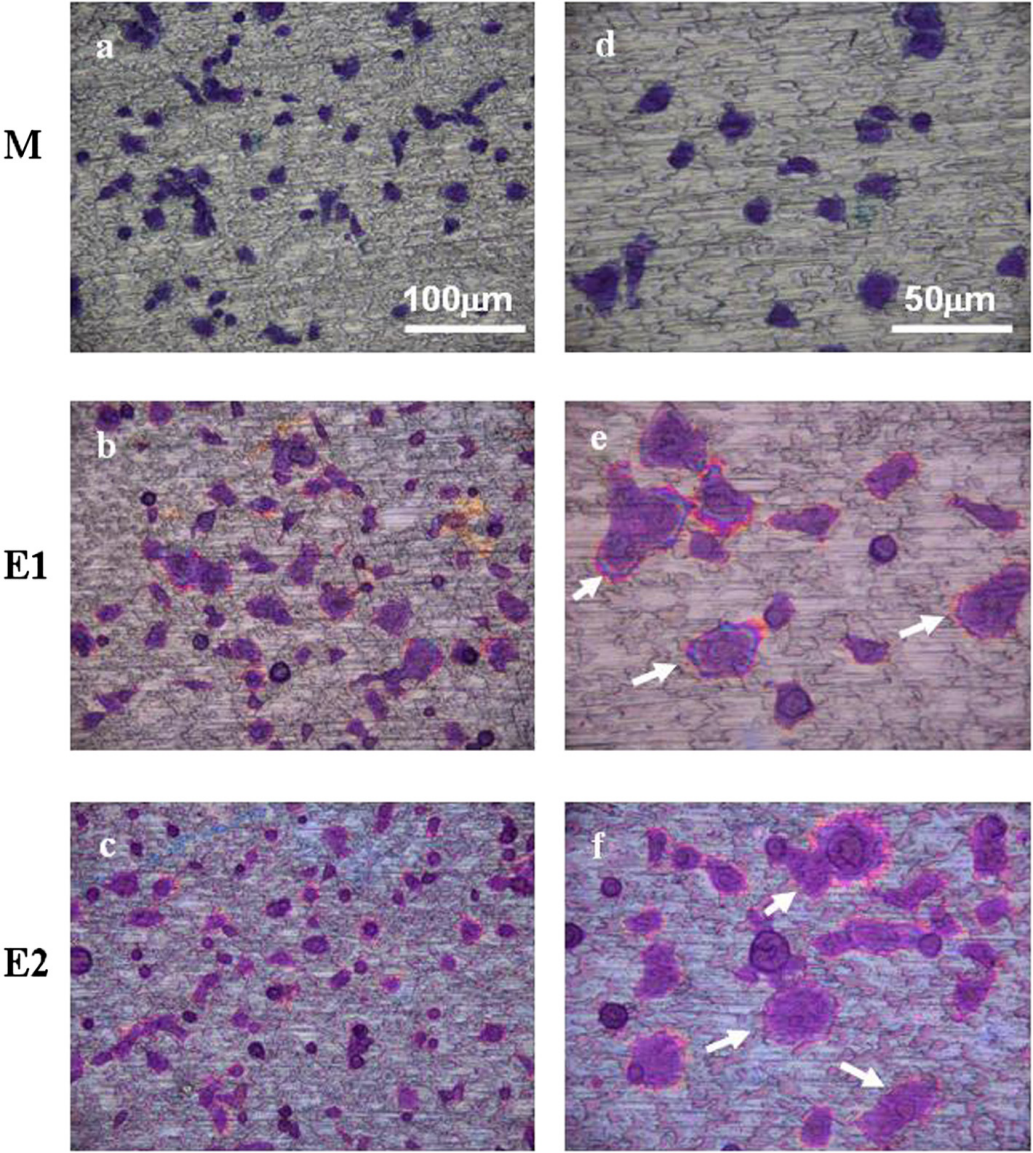
**Crystal violet staining micrographs after 12 h of cell incubation.** Crystal violet staining micrographs of HNEpC on the Ti specimens (M, E1, and E2) after 12 h of cell incubation (**a**, **b**, and **c**: M, E1, and E2 at low magnification, respectively; **d**, **e**, and **f**: M, E1, and E2 at high magnification, respectively) (arrow: spreading cells).

**Figure 5:**
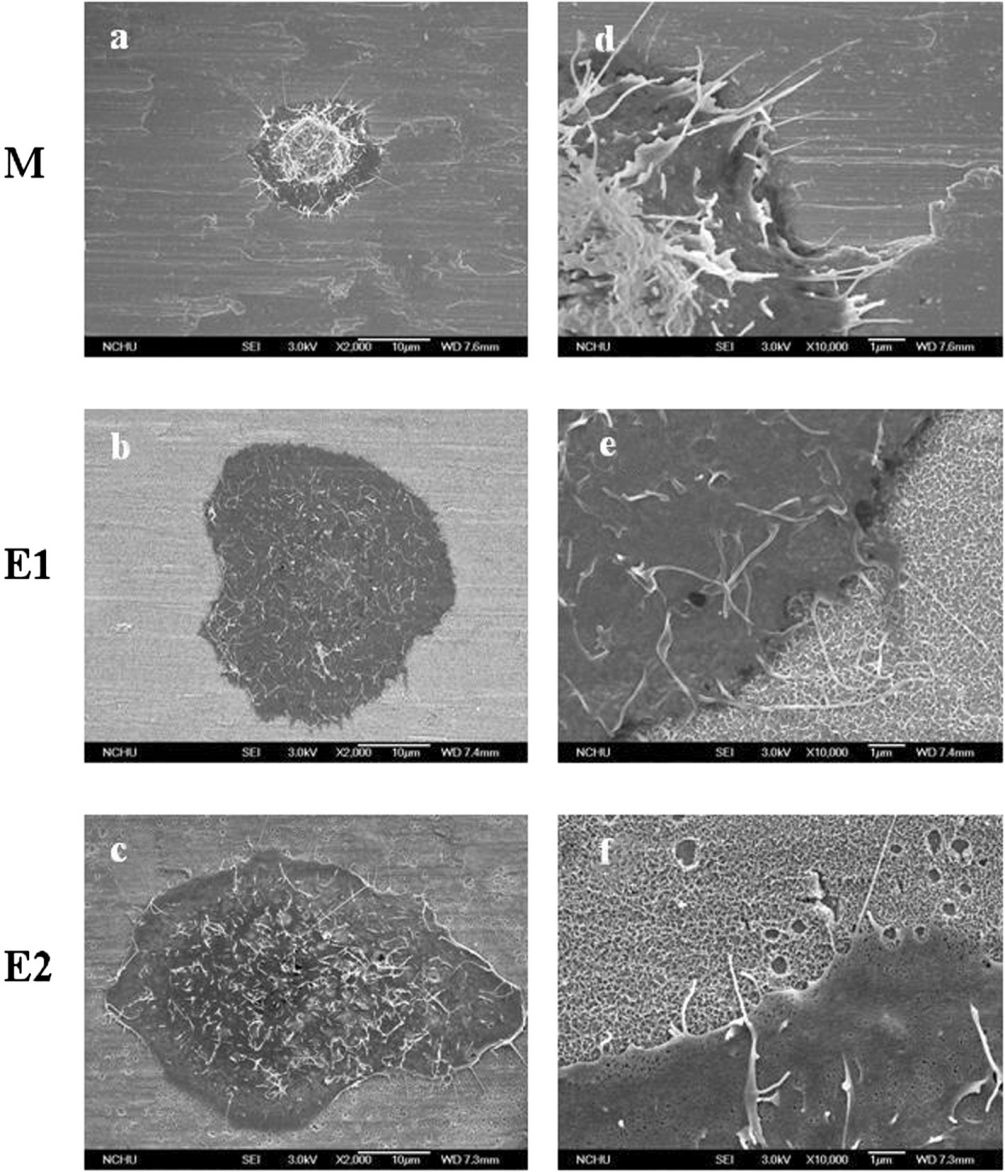
**FE-SEM micrographs after 12 h of cell incubation.** FE-SEM micrographs of HNEpC on the Ti specimens (M, E1, and E2) after 12 h of cell incubation (**a**, **b**, and **c**: M, E1, and E2 at × 2,000 magnification, respectively; **d**, **e**, and **f**: M, E1, and E2 at × 10,000 magnification, respectively).

**Figure 6:**
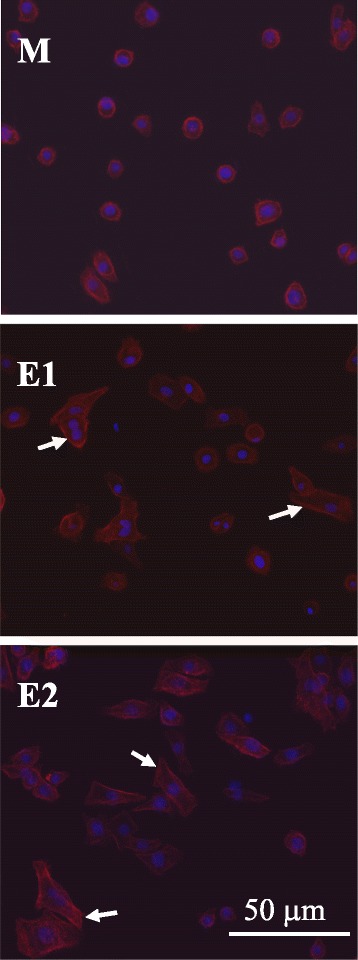
**Cell adhesion images, using immunofluorescent staining, after 12 h of cell incubation.** Cell adhesion images, using immunofluorescent staining, of HNEpC on the Ti specimens (M, E1, and E2) after 12 h of cell incubation: dual staining of DAPI for nuclei (blue color) and rhodamine phalloidin for actin filaments (red color) (arrow: spreading cells).

Crystal violet staining, FE-SEM micrographs, and immunofluorescent staining of cells attached to the Ti specimens (M, E1, and E2) after 12 h of cell incubation are presented in Figures 4, 5, and 6, respectively. As shown in Figure 4, the cells on the anodized E1 and E2 specimens have already entered the spreading phase. The anodized E1 and E2 specimens with a nanoscale structure may play an important role in the stimulation of cell attachment initiation. Similar results were also observed in the FE-SEM micrographs (Figure 5) and immunofluorescent staining (Figure 6). HNEpC on the anodized E1 and E2 specimens typically exhibited epithelial morphology, and the lamellipodia and filopodia revealed improved cell-substrate interaction compared with specimen M (Figure 5). Immunofluorescent staining was used to investigate the cells adhesion to the Ti specimens and also revealed enhanced spreading and a cytoskeletal arrangement of the cells on the anodized E1 and E2 specimens (Figure 6). The improved adhesion appearance of HNEpC observed on the anodized Ti specimens was a positive effect of biocompatibility.

**Figure 7:**
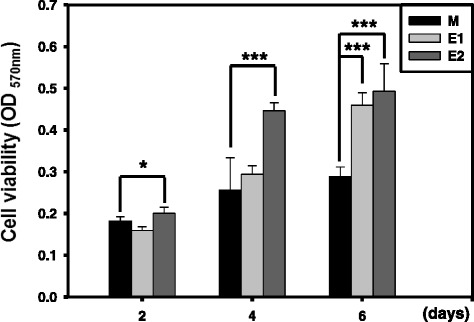
**Proliferation of HNEpC on the Ti specimens during 6 days of cell incubation.** Proliferation of HNEpC on the Ti specimens (M, E1, and E2) during 6 days of cell incubation: data are reported as the mean and standard deviation (*p < 0.05 and ***p < 0.001). Indicating a significant difference for the E1 and E2 specimens compared with the M specimen.

The proliferation of HNEpC on the Ti specimens was examined using the MTT assay, in terms of cell viability, at 2, 4, and 6 days, as illustrated in Figure 7. During the 6 days of incubation, the number of cells on the anodized E1 and E2 specimens was significantly higher than that on the M specimen; moreover, the E2 specimen exhibited faster cell growth than the E1 specimen at day 4. These results suggest that the Ti surface with a hydrophilic nanonetwork structure can promote HNEpC proliferation.

Cell attachment and spreading are the initial phase of cytocompatibility, and the quality of this phase influences the capacity for cell proliferation. It is well known that surface characteristics of biomaterials play an important role in the cell responses. As for hydrophilicity, most reports found that the hydrophilic surfaces are liable to improve the cell adhesion and proliferation [37-39]. Some opposite results have been also reported [40,41]. On the other hand, no correlation is observed between the surface hydrophilicity and cell adhesion [42,43]. In this study, the anodized E1 and E2 specimens with super hydrophilic surface (PBS contact angle <5°) revealed better HNEpC attachment, spreading, and proliferation than the non-anodized M specimens. As for surface nanotopography, a previous study has demonstrated that nanoscale surfaces can stimulate cell responses without exposure to any molecular signals [44]. Similar results have been reported that the pore sizes of 15 to 80 nm of the self-assembled TiO_2_ nanotube surfaces are beneficial for cell adhesion [45-47]. In this study, the electrochemical anodization treatment appears not to have changed the surface topography of the M specimens at the microscale but rather produced a unique hydrophilic nanonetwork structure with average pore size of approximately 70 nm and 90 nm on the anodized E1 and E2 specimens, respectively. This degree of surface porosity did not significantly affect the surface roughness *R*_a_ of the specimens, despite the fact that the thin anodized surface layer was 200 or 300 nm in thickness. Positive effects on cell responses, including cell attachment, spreading, and proliferation, of HNEpC were observed for a range of pore sizes (10 to 150 nm) in the anodized specimens (Figures 4, 5, 6, and 7). The TiO_2_ nanonetwork surface provides a suitable substrate for cell growth and thus enhances various cell behaviors, such as cell adhesion, spreading, and proliferation.

## Conclusions

A unique super hydrophilic TiO_2_ nanonetwork structure, with lateral pore sizes of 10 to 150 nm, was rapidly produced on a Ti surface using an electrochemical anodization treatment. Based on the results of this study, anodized Ti surfaces with hydrophilic nanonetwork topography is a potential candidate for achieving favorable cell responses of primary HNEpC. The hydrophilic TiO_2_ nanonetwork structure on the Ti surface enhanced protein adsorption, cell adhesion, cell spreading, cytoskeletal arrangement, and cell proliferation. This research provided advanced information on the positive effect of a hydrophilic TiO_2_ nanonetwork layer on the response of HNEpC to Ti surfaces for potential nasal implant applications.
